# Roles of *GFAT* and *PFK* genes in energy metabolism of brown planthopper, *Nilaparvata lugens*

**DOI:** 10.3389/fphys.2023.1213654

**Published:** 2023-06-21

**Authors:** Hui-Ru Si, Si-Si Sun, Yong-Kang Liu, Ling-Yu Qiu, Bin Tang, Fang Liu, Qiang Fu, Cai-Di Xu, Pin-Jun Wan

**Affiliations:** ^1^ State Key Laboratory of Rice Biology and Breeding, China National Rice Research Institute, Hangzhou, Zhejiang, China; ^2^ College of Life and Environmental Sciences, Hangzhou Normal University, Hangzhou, Zhejiang, China; ^3^ Guizhou Institute of Mountainous Environment and Climate, Guiyang, China; ^4^ Jing Hengyi School of Education, Hangzhou Normal University, Hangzhou, Zhejiang, China

**Keywords:** *Nilaparvata lugens*, RNA interference, glutamine: fructose-6-phosphate aminotransferase, phosphofructokinase, energy metabolism

## Abstract

Glutamine:fructose-6-phosphate aminotransferases (GFATs) and phosphofructokinase (PFKs) are the principal rate-limiting enzymes involved in hexosamine biosynthesis pathway (HBP) and glycolysis pathway, respectively. In this study, the *NlGFAT* and *NlPFK* were knocked down through RNA interference (RNAi) in *Nilaparvata lugens*, the notorious brown planthopper (BPH), and the changes in energy metabolism were determined. Knockdown of either *NlGFAT* or *NlPFK* substantially reduced gene expression related to trehalose, glucose, and glycogen metabolism pathways. Moreover, trehalose content rose significantly at 72 h after ds*GFAT* injection, and glycogen content increased significantly at 48 h after injection. Glucose content remained unchanged throughout the experiment. Conversely, ds*PFK* injection did not significantly alter trehalose, but caused an extreme increase in glucose and glycogen content at 72 h after injection. The Knockdown of *NlGFAT* or *NlPFK* significantly downregulated the genes in the glycolytic pathway, as well as caused a considerable and significant decrease in pyruvate kinase (PK) activity after 48 h and 72 h of inhibition. After ds*GFAT* injection, most of genes in TCA cycle pathway were upregulated, but after ds*NlPFK* injection, they were downregulated. Correspondingly, ATP content substantially increased at 48 h after *NlGFAT* knockdown but decreased to an extreme extent by 72 h. In contrast, ATP content decreased significantly after *NlPFK* was knocked down and returned. The results have suggested the knockdown of either *NlGFAT* or *NlPFK* resulted in metabolism disorders in BPHs, highlighting the difference in the impact of those two enzyme genes on energy metabolism. Given their influence on BPHs energy metabolism, developing enzyme inhibitors or activators may provide a biological control for BPHs.

## 1 Introduction

Insects depend on a constant intake of energy to maintain physiological processes, and energy metabolism occures throughout their body. Unlike mammals, insects primarily utilize trehalose for energy instead of glucose (Yasugi et al., 2017). Trehalose is a disaccharide that serves as the fundamental carbohydrate constituent of hemolymph, and is highly stable. It is found in various organisms, including bacteria, yeast, fungi, nematodes, insects, invertebrates, and plants, but is absent in mammals (Elbein et al., 2003; Wen et al., 2016; Wang et al., 2020). Trehalose plays crucial role in acclimating to various abiotic stresses, such as high or low temperatures, nutrition or starvation, oxidation stress, high osmotic pressure, toxic substances, and UV-B irradiation (Tamang et al., 2017; Chen et al., 2018). Moreover, trehalose can serve as an instant source of energy. Trehalose-6-phosphate synthase (TPS) is responsible for the conversion of uridine diphosphate glucose (UDP-glucose) and glucose-6-phosphate (G-6-P) to trehalose-6-phosphate, which then undergoes dephosphorylation by trehalose-6-phosphate phosphatase (TPP) to produce trehalose. This pathway of trehalose synthesis is the most important in insects known to date (Shukla et al., 2015; Tang et al., 2017; Chen et al., 2018).

Trehalase (TRE) is the sole disaccharidase that degrades trehalose in insects, catalyzing its conversion to glucose when energy is needed (Barraza and Sánchez, 2013; Luo et al., 2022). The resulting glucose is utilized in the glycolysis-tricarboxylic acid (glycolysis-TCA) cycle, which converts glucose into pyruvate via a series of enzymatic reactions. These reactions involve hexokinase (HK), glucose-6-phosphate isomerase (G6PI), phosphofructokinase (PFK) and pyruvate kinase (PK) (Hu et al., 2016; Hassan et al., 2022). The reverse process of glycolysis is gluconeogenesis, which is accomplished fructose-1,6-bisphosphatase (FBP). Pyruvate is then converted into acetyl-coenzyme A (acetyl-CoA) which combines with oxaloacetic acid to enter TCA cycle, resulting in the generation of multiple adenosine triphosphate (ATP) (Hu et al., 2016). Insects have the ability to regulate the rate of glycolysis-TCA cycle to accommodate various physiological activities. For example, *Drosophila melanogaste*, *Bombyx mori*, and *Spodoptera litura*, decrease the relative expression of genes encoding glycolysis or TCA metabolic enzymes during early pupal development in order to conserve energy for organogenesis (White et al., 1999; Tian et al., 2010; Hu et al., 2016).

In addition to being converted into trehalose, glucose from the environment in insects can also converted into glycogen, which is another critical metabolic and energy substance (Tolmasky et al., 2001; Liu et al., 2009). Glycogen is synthesized and stored in the fat body, and then can be quickly converted into glucose or trehalose and transported to other tissues (Tang et al., 2012). The synthesis and degradation of glycogen molecules are regulated by the activity of enzymes, primarily by glycogen synthase (GS) and glycogen phosphorylase (GP), respectively (Prats et al., 2005). GS use UDP-glucose as its sole substrate to synthesize glycogen from glucose monomers, while GP breaks down the glycogen. Notably, UDP-glucose is also critical for the synthesis of trehalose in insects (Tang et al., 2012). During high-energy behaviors like flight, insects transfer trehalose from their hemolymph to their flight muscles for instant energy supply. Therefore, glycogen stored in the fat body must be converted into trehalose to maintain sufficient hemolymph trehalose concentration (Yu et al., 2008). Furthermore, some insects, such as the ladybird *Coccinella septempunctata* Linnaeus (Ren et al., 2015) and *Zygaena trifolii* (Esper) (Lepidoptera: Zygaenidae), accumulate adequate glycogen in preparation for entering diapause (Wipking et al., 1995).

Fructose-6-phosphate (F-6-P) is produced by G6PI catalyzing the isomerization of G-6-P and is an important intermediate substance in the conversion process of trehalose, glucose, and glycogen, which is crucial in energy metabolism (Achari et al., 1981). F-6-P enters various metabolic pathways, depending on enzymatic conversion. One pathway for F-6-P is conversion into fructose-1,6-diphosphate, an important intermediate in glycolysis that is catalyzed by phosphofructokinase (PFK). PFK is a highly conserved enzyme in the glycolytic pathway and is the main rate-limiting enzyme. The activity of PFK has a significant impact on both glucose consumption and energy production (Jojima and Inui, 2015). PFK in vertebrates is activated by ADP, AMP, and fructose-2,6-diphosphate, while it is inhibited by physiological levels of ATP and citrate (Martínez-Costa et al., 2004). However, PFK in insect does not be inhibited by citrate (Newsholme et al., 1977; Nunes et al., 2016). Another pathway for F-6-P is to generate glucosamine-6-phosphate under the action of glutamine:fructose-6-phosphate aminotransferase (GFAT) and participate in hexosamine biosynthesis pathway (HBP) to chitin production (Zhu et al., 2016). Chitin is a linear polymer composed of N-acetylglucosamine units linked by β-1,4-glycoside bonds and it is a major component of exoskeleton, trachea and the peritrophic matrix that lines the midgut epithelium (Merzendorfer and Zimoch, 2003; Tharanathan and Kittur, 2003; Xi et al., 2015; Liu et al., 2019). Since GFAT is the first and rate-limiting enzyme of HBP, it significantly impacts on chitin synthesis (Denzel and Antebi, 2015). Overall, PFK and GFAT are two crucial enzymes in the energy metabolism pathway of insects.

*Nilaparvata lugens*, commonly known as the brown planthopper (BPH), is one of the most destructive and notorious rice pests in Asia and responsible for serious crop losses (Zhou et al., 2018; Li et al., 2021). BPHs have high fecundity and feed on rice sap, oviposit in rice tissues, and transmit viruses such as grassy stunt virus and rugged stunt virus (Sun et al., 2017; Bing et al., 2019). The BPH is not only abundant, but it is also prone to developing insecticide resistance easily (Mu et al., 2016). Therefore, it is difficult to manage BPH with standard chemical methods and should be manage through natural enemies (Liu et al., 2022). In this study, RNA interference (RNAi), a commonly used method for studying of insect gene function (Ullah et al., 2022; Zhou et al., 2022), was employed to suppress the expression of *NlGFAT* and *NlPFK* genes. This analysis revealed the impacts of these two enzymes on insect energy metabolism, opening up new opportunities for pest control.

## 2 Materials and methods

### 2.1 Experimental insects and material collection

The BPH colonies used in this experiment were provided by the China National Rice Research Institute (Hangzhou, China), and were kept in laboratory for at least 30 generations. The rice (*Oryza sativa* L.) used in this study was Taichung Native 1 (TN1) and was grown in cement tanks (60 cm × 30 cm  ×  100 cm). Insects were reared on fresh rice seedlings in an artificial climate chamber at 26°C ± 1°C, 70% relative humidity, and 16L:8D (light: dark) photoperiod.

The microinjected insects were first day, fifth instar nymph BPH. Each treatment comprised 240 nymphs divided into 12 replicates of 20 individuals per replicate. Three replicates were used for each of total RNA isolation, sugar content determination, and enzyme activity determination, while one additional replicate was used for ATP content determination. Insects were collected at 48 h and 72 h after injection and stored at −80°C for determination of gene expression, sugar content, enzyme activity, and ATP content. All experiments were independently conducted three times for biological replication.

### 2.2 Total RNA isolation and cDNA synthesis

Total RNA was extracted using Trizol from five randomly selected individuals for each treatment (Invitrogen, Carlsbad, California, United States), following the manufacturer’s instructions. RNA integrity was determined with 1% agarose gel electrophoresis, and RNA concentration and purity were assessed with a Nanodrop 2,000 spectrophotometer (Thermo Fisher Scientific, Waltham, MA, United States) by measuring absorbance at 260 nm. Purified RNA was stored at −80°C for subsequent experiments. First-strand complementary DNA (cDNA) was synthesized using the PrimeScript RT reagent kit with gDNA Eraser (Takara, Kyoto, Japan) following the manufacturer’s instructions and stored at −20°C.

### 2.3 Double-stranded RNA (dsRNA) synthesis and injections

The dsDNA fragments from *NlGFAT* (OR058797) and *NlPFK* (OR058799) genes were amplified by PCR using specific primers containing the T7 promoter sequence at their 5′ ends (Table 1). The PCR amplification was carried out under the following conditions: preincubation at 95°C for 3 min, 35 cycles at 95°C for 30  s, 55°C for 30 s, 72°C for 1 min, and a last extension at 72°C for 10 min. The purified amplification products of *NlGFAT* and *NlPFK* were used to synthesize dsRNA by *in vitro* transcription using T7 RiboMax Express RNAi System (Promega, Madison, WI). The dsRNA obtained from green fluorescence protein (*GFP*) gene was used as a control. The sense and anti-sense strands were first produced in two separate transcription procedures and then mixed for annealing. The reaction mixture was incubated at 70°C for 10 min and then cooled on an ice bath for 20 min. The dsRNAs were then precipitated with 95% ethanol and 3 M sodium acetate (pH 5.2), washed with 70% ethanol, air dried, and resuspended. The integrity and quantity of dsRNAs were evaluated by spectroscopy analysis with Nanodrop 2,000 and by 1% agarose gel electrophoresis (Zhang et al., 2017). The synthesized dsRNA was stored at −80°C.

**TABLE 1:** Gene-specific primers used for double-stranded RNA synthesis.

Primer	Forward primer (5′–3′)	Reverse primer (5′–3′)
dsNlGFAT-F	GCC​TGA​TGC​TGA​TTG​GGT​G	CGA​GAT​GAA​CTG​GGA​GGT​GTA​G
dsNlGFAT-T7	T7- GCC​TGA​TGC​TGA​TTG​GGT​G	T7-CGA​GAT​GAA​CTG​GGA​GGT​GTA​G
dsNlPFK-F	AGATCGTTCGTCCGCAAC	CCC​GCT​AGA​CCA​GCA​ATA​GTA
dsNlPFK-T7	T7-AGA​TCG​TTC​GTC​CGC​AAC	T7-CCC​GCT​AGA​CCA​GCA​ATA​GTA
dsGFP-F	AAG​GGC​GAG​GAG​CTG​TTC​ACC​G	CAG​CAG​GAC​CAT​GTG​ATC​GCG​C
dsGFP-T7	T7-AAG​GGC​GAG​GAG​CTG​TTC​ACC​G	T7-CAG​CAG​GAC​CAT​GTG​ATC​GCG​C
T7 sequence: 5′- GGA​TCC​TAA​TAC​GAC​TCA​CTA​TAG​G -3′		

The abdomen of each BPH (on 1st day of the 5th instar nymphs) between the second pair of peids and the third pair of pedis was injected with 3,000 ng of ds*GFAT* and ds*PFK* (of each) using an IM-31 microinjector (NARISHIGE, Tokyo, Japan). The control groups were injected with ds*GFP*. As the interference efficiency of the same dsRNA was previously determined in the study (Xu et al., 2021), it was not determined separately in current study.

### 2.4 Quantitative real-time polymerase chain reaction (qRT-PCR)

The total RNA of BPHs collected after dsRNA injection were extracted and reverse transcribed into cDNA, which was used as a template. Specific primers were selected (Table 2). The relative expression of genes was estimated by qRT-PCR with a SYBR Green master mix (Takara) in a CFX96TM Real-Time PCR Detection System (Bio-Rad, Hercules, CA, United States). Each PCR was performed in a 20 μL volume, containing 1 μL cDNA, 1 μL (10 µM) each primer, 7 μL ultrapure water, and 10 μL SYBR buffer. The reaction was performed with following conditions: preincubation at 95°C for 2 min, followed by 39 cycles of 95°C for 5 s and annealing at 59°C for 30 s, with a melting curve at 65–95°C. Amplification of 18 S RNA was used as an internal control. The 2^−△△CT^ method was used for the analysis of relative gene expression (Livak and Schmittgen, 2001).

**TABLE 2:** Gene-specific primers used for quantitative real-time polymerase chain reaction (qRT-PCR).

Primer name	Forward primer (5′–3′)	Reverse primer (5′–3′)
*QNl18S*	CGCTACTACCGATTGAA	GGA​AAC​CTT​GTT​ACG​ACT​T
*QNlTPS1*	AAGACTGAGGCGAATGGT	AAG​GTG​GAA​ATG​GAA​TGT​G
*QNlTPS2*	AGA​GTG​GAC​CGC​AAC​AAC​A	TCA​ACG​CCG​AGA​ATG​ACT​T
*QNlTPS3*	GTG​ATG​CGT​CGG​TGG​CTA​T	CCG​TTC​ATC​ATT​GGG​CAT​AGT
*QNlTRE1-1*	GCCATTGTGGACAGGGTG	CGG​TAT​GAA​CGA​ATA​GAG​CC
*QNlTRE1-2*	GATCGCACGGATGTTTA	AATGGCGTTCAAGTCAA
*QNlTRE2*	TCACGGTTGTCCAAGTCT	TGTTTCGTTTCGGCTGT
*QNlHK*	GGT​GCG​AGA​AGA​AGT​GAA​G	GTG​AAA​CCC​ATT​GGT​AGA​GT
*QNlG-6-pase*	TTTCGGCTCACTTCCCTC	GCA​GTA​ATC​AAC​ATA​GCA​CCT
*QNlUGPase*	GCACGGTGACTTCTACGA	TGAGGTCAACTGTGGCTC
*QNlGP*	GCT​GCC​TAT​GGC​TAT​GGT​ATT​C	TCT​GAG​TGT​TGA​CCC​ACT​TCT​TG
*QNlGS*	GCT​CCA​AAG​CCT​ATG​TTT​CTA​CTG	TGG​TAA​CCC​CTG​TCC​CTC​A
*QNlG6PI1*	GTT​CAC​GGT​CGT​CTG​GAA​AG	TGA​CTG​CTC​CGT​TTC​ACT​CT
*QNlG6PI2*	AAC​AAG​GCG​ACA​TGG​AAT​CG	ACC​ATT​TGT​TCC​TGG​TTC​GC
*QNlG6PI2*	ATG​TCA​CAG​TGC​ATG​TCG​TG	ACC​TGC​TCT​CAT​TGA​TGC​CA
*QNlPFK*	TGACGTGACAGGGTGGGT	ATG​GCT​TGG​ATT​TGG​AAC​T
*QNlPK1*	ATG​ATA​ACG​GGT​CAG​GCG​AT	TAC​CGA​ACC​ACC​GAA​GAA​CA
*QNlPK2*	TCC​CGA​CTA​TGA​CCT​TGC​TC	AGT​GAC​CAC​CAA​ACC​AAA​CG
*QNlPK3*	AGA​AGA​AGA​CAT​GCC​GCA​AC	TCG​TGA​GTT​GAG​TGA​GCC​AT
*QNlPK4*	ATC​CGG​ACA​CCA​ACA​CTC​TT	ACA​AAC​TGG​TCG​CTT​TCA​CC
*QNlPK5*	TGG​TCT​AGC​CTG​GAC​TGT​TG	AGC​TTT​CCC​TCT​GCA​TCC​AT
*QNlPK6*	GTG​CTA​CAG​ATC​GAC​CCA​GA	GGT​TTG​GCA​GCT​TGA​CTG​AA
*QNlPK7*	AAG​GGA​ACC​GTT​CAC​AGC​TA	TGT​CTT​TCT​CGC​CTG​TCA​CT
*QNlPK8*	ATC​CGG​ACA​CCA​ACA​CTC​TT	CCC​TCC​ACC​GGG​ATT​ATG​AA
*QNlPK9*	CAA​TTC​GGC​AGT​GGC​ATA​CA	TGG​ACA​TGA​GCT​TCA​CCA​CT
*QNlPK10*	ACC​TGT​GGA​TCA​GAC​TGC​AA	CTG​ATG​CTC​CTG​ACG​TTG​TG
*QNlAH1*	CGA​GAC​GAT​CAT​TGC​TGG​TG	TAT​TGT​CAG​CGT​CGC​AAA​GG
*QNlAH2*	GCG​ACG​TGG​ACA​ATG​TGT​TA	TCT​GGA​TTT​CCA​CCC​AGG​TC
*QNlMDH1*	GCG​ATC​CTG​TCT​CAT​TGA​CG	TGG​CCA​TAG​TAG​GGC​TTG​AC
*QNlMDH2*	AGG​CTG​GAA​CTA​AGG​TGG​TC	GCA​GAA​CAG​CTG​TCG​AGA​AG
*QNlMDH3*	ACT​ACA​AGC​CCA​GTC​AGC​AT	TGC​TGT​GTC​CAA​CTC​CAG​AT
*QNlMDH4*	CGA​GGA​TGA​CGA​CGA​TGA​TG	TCC​AGT​TTG​GGT​GGA​CTC​TC
*QNlMDH5*	TTG​CTG​CTC​AAC​CAG​TGA​AC	TTC​AAT​GTG​AAG​CCG​ACC​AC

### 2.5 Determination of TRE activity and sugar content

TRE in BPH is classified into soluble trehalase (TRE1) and membrane-bound trehalase (TRE2). Thirty BPH individuals collected after dsRNA injection were homogenized in 200 μL phosphate-buffered saline (PBS; pH 7.0), and then mixed with 800 μL of PBS were added. Subsequently, the homogenate was centrifuged at 1,000 g for 20 min at 4°C. The supernatant (300 μL) was taken to detect concentration of protein, trehalose and glycogen and the resting the supernatant (350 μL) was removed and ultracentrifuged at 20,800 g for 60 min at 4°C. The supernatant (300 μL) obtained from ultracentrifugation was used to determine TRE1 activity and concentration of protein and glucose. The sediment was suspended in PBS (300 μL) and was used for the determination of TRE2 activity and concentration of protein and glucose.

The previous method with proper modifications was used to TRE activity assay (Tatun et al., 2008a; Tatun et al., 2008b). Anthrone method was used to determination of trehalose (Zhang et al., 2017). The glucose content was determined by glucose assay (Sigma-Aldrich, St. Louis, MO, United States). The glycogen content was also determined by glucose assay after converting to glucose under the action of amyloglucosidase (Sigma-Aldrich) (Yang et al., 2017). The protein concentration was determined to calculate the content of glycogen, trehalose and glucose, following the BCA Protein Assay Kit (Beyotime, Shanghai, China) according to the manufacturer’s instruction.

### 2.6 Measurement of activity of PK and MDH and ATP content

The BPHs were mixed with 1× PBS for grinding and crushing to obtain 10% homogenate, and the experiment was carried out according to the instruction of Pyruvate Kinase Assay Kit (Jiancheng, Nanjing, China) and Malate Dehydrogenase Assay Kit (Jiancheng). For ATP content measurement, it was measured following ATP Aassay Kit (Jiancheng).

### 2.7 Statistical analysis

The data were presented as the mean ± standard error (SE). After respectively testing for normality (Shapiro–Wilk test) and homogeneity variance (Levene’s tests), these data were further evaluated by a two-way analysis of variance (ANOVA) following by Dunnett’s *post hoc* test to determine the differences across various treatments. The difference was considered as significant or extremely significant when the *p*-value below 0.05 or 0.01, respectively.

## 3 Results

### 3.1 Relative expression of genes in carbohydrate conversion pathway

**FIGURE 1:**
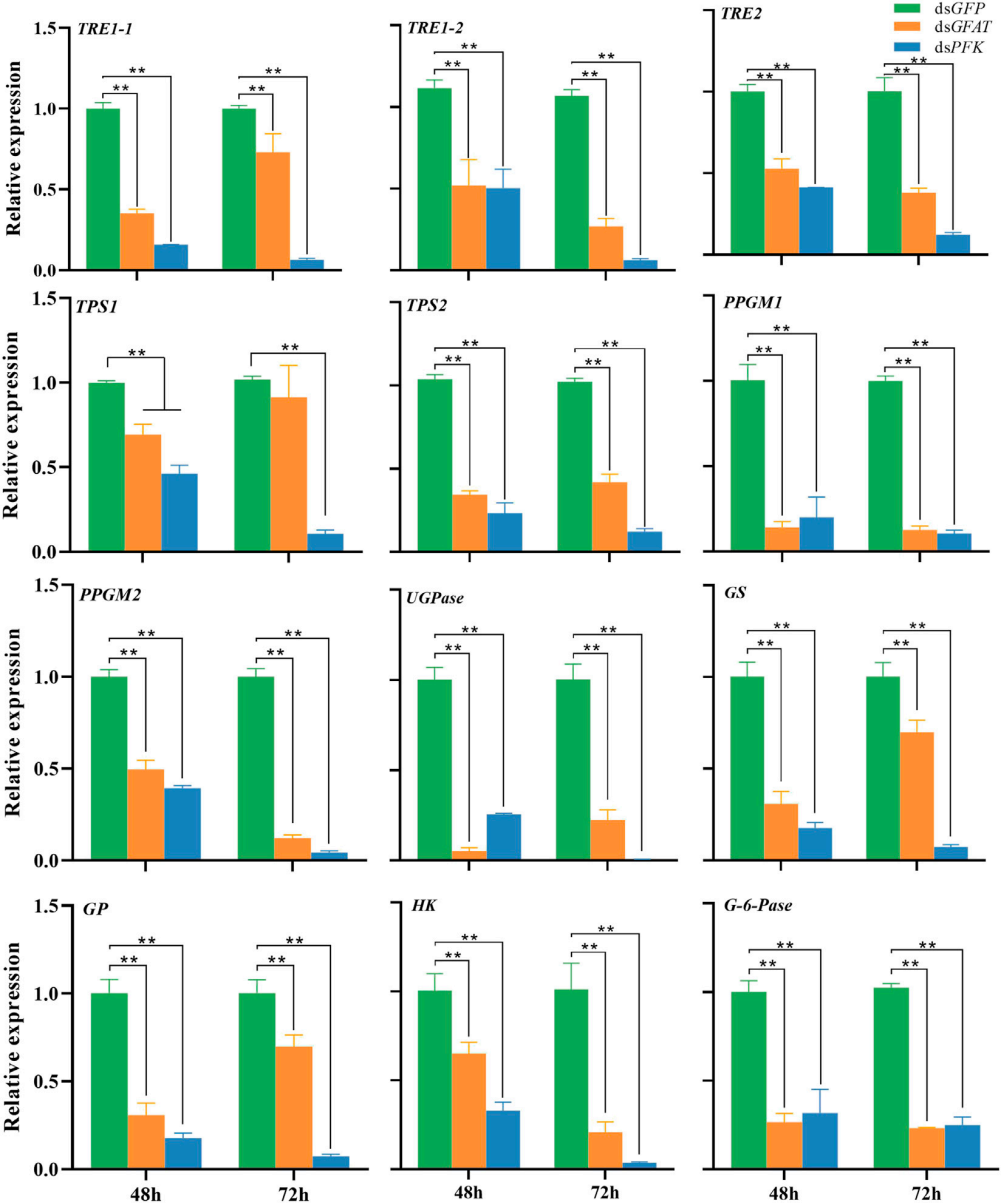
Relative expression of genes in carbohydrate conversion pathway at 48 h and 72 h after dsRNA injection. The qRT-PCR is used to detect the genes expression levels and the 18 s RNA is used as internal control. Bars are means ± SE (standard error) of three biological replicates. An asterisk (*) represents significant differences (*p* < 0.05); two asterisk (**) represents extremely significant differences (*p* < 0.01). TRE, trehalase; TPS, trehalose-6-phosphate synthase; PPGM, phosphoglucomutase; UGPase, UDP-Glucose pyrophosphorylase; GS, glycogen synthase; GP, glycogen phosphorylase; HK, hexokinase; G-6-pase, glucose-6-phosphatase.

The qPCR results have shown that the mRNA levels of *TRE1-1*, *TRE1-2*, *TRE2*, *HK*, *PPGM1*, *PPGM2*, *UGPase*, and *TPS2* were significantly decreased at 48 h and 72 h after inhibition of *GFAT* or *PFK* (Figure 1). The expression levels of *TPS1* were significantly decreased following ds*PFK* injection at 48 h and 72 h (Figure 1), while its mRNA level was downregulated at 48 h but return to the same level as the control group at 72 h after ds*GFAT* injection. Both gluconeogenesis and glycogenolysis result in the formation of G-6-P, which is hydrolyzed to glucose by G-6-pase (van and Gerin, 2002). *G-6-pase* was significantly downregulated after *GFAT* or *PFK* was knocked down (Figure 1). However, when *GFAT* or *PFK* was inhibited, the relative expressions of *GS* and *GP* were significantly decreased (Figure 1). These results suggest that both GS and GP genes, as well as other genes in the carbohydrate conversion pathway, were downregulated after *GFAT* or *PFK* was knocked down, respectively.

### 3.2 Content of trehalose, glucose and glycogen after dsRNA injection

**FIGURE 2:**
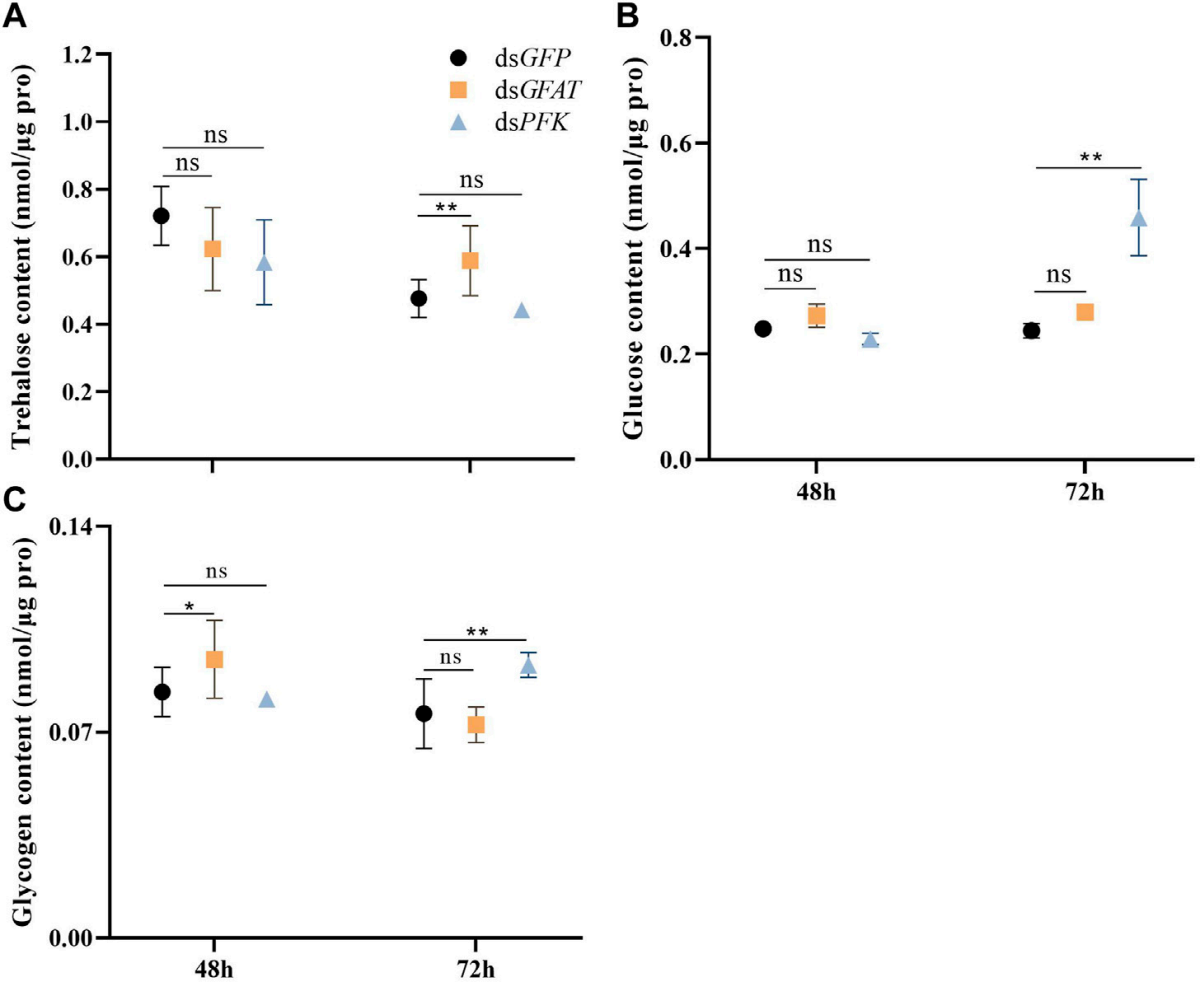
Content of trehalose, glucose and glycogen at 48 h and 72 h after dsRNA injection. *Nilaparvata lugens* on 1st of 5th instar were used to inject. Bars are means ± SE (standard error) of three biological replicates. An asterisk (*) represents significant differences (*p* < 0.05); two asterisk (**) represents extremely significant differences (*p* < 0.01). ns: not significant.

The content of trehalose remained consistent after 48 h and subsequently decreased significantly at 72 h following the injection of ds*GFAT* (Figure 2A). Conversely, the glycogen content increased significantly at 48 h before returning to normal levels at 72 h following the injection of ds*GFAT* (Figure 2C). In contrast, there was no significant change in glucose content following the injection of ds*GFAT* (Figure 2B). Inhibition of the *PFK* gene resulted in the maintenance of trehalose content (Figure 2A), while both glucose and glycogen content showed no significant change at 48 h but increased significantly at 72 h (Figure 2C).

### 3.3 Relative expression of crucial enzyme genes in glycolytic pathway

**FIGURE 3:**
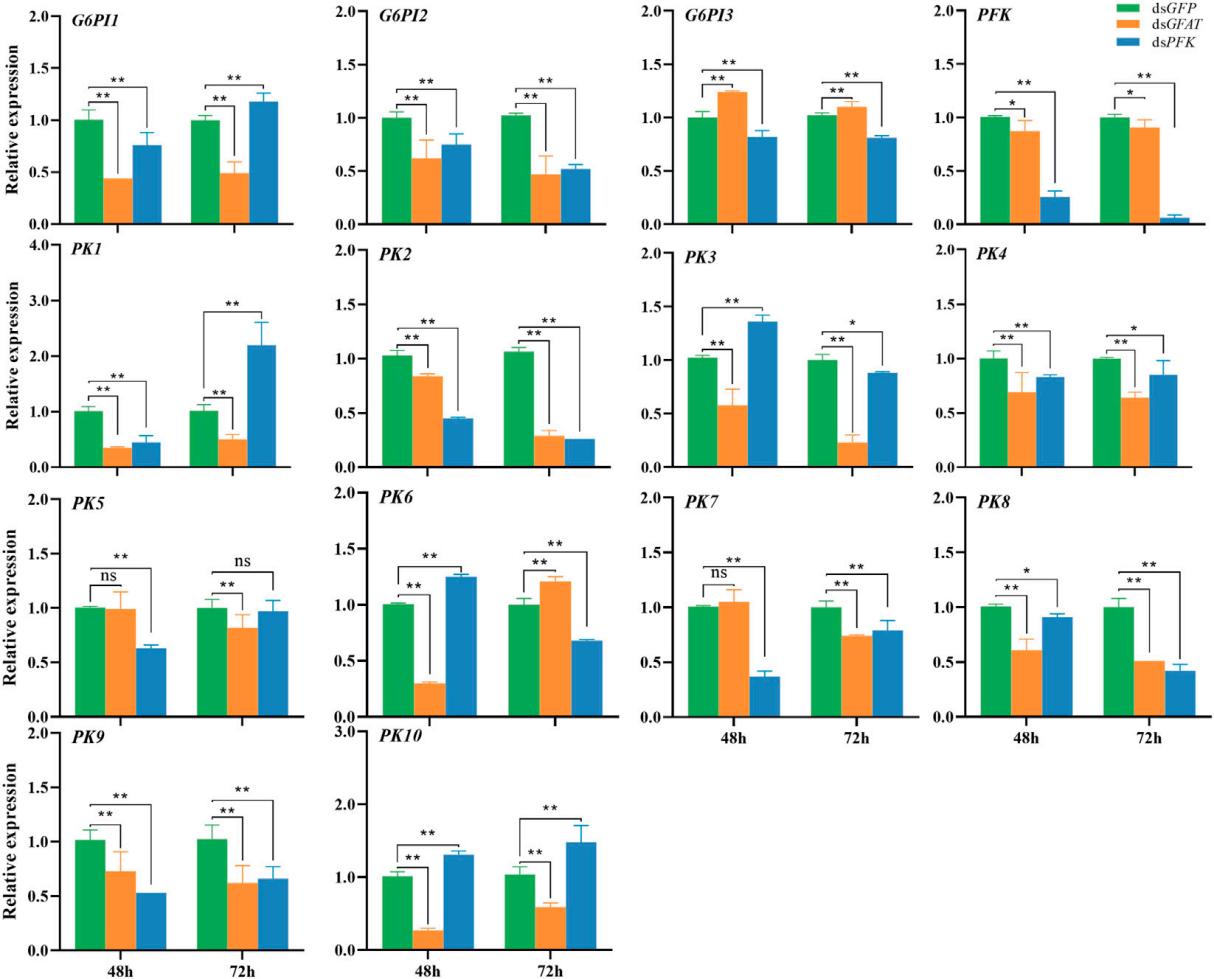
Relative expression of crucial genes in glycolytic pathway at 48 h and 72 h after dsRNA injection. The qRT-PCR is used to detect the genes expression levels and the 18 s RNA is used as internal control. Bars are means ± SE (standard error) of three biological replicates. An asterisk (*) represents significant differences (*p* < 0.05); two asterisk (**) represents extremely significant differences (*p* < 0.01). G6PI, glucose-6-phosphate isomerase; PFK, phosphofructokinase; PK, pyruvate kinase.

After *GFAT* was knocked down, the relative expression of *G6PI1*, *G6PI2*, *PFK*, *PK1*, *PK2*, *PK3*, *PK4*, *PK8*, *PK9*, and *PK10* were downregulated both at 48 h and 72 h (Figure 3). The relative expression of *G6PI3* was sharply upregulated (Figure 3), while *PK5* and *PK7* expression had no significance change at 48 h but decreased significantly at 72 h (Figure 3), and the mRNA level of *PK6* extremely reduced at 48 h but increased significantly at 72 h (Figure 3). After *PFK* was knocked down, the relative expression of *G6PI2*, *G6PI3*, *PFK*, *PK2*, *PK4*, *PK7*, *PK8*, and *PK9* decreased significantly (Figure 3). The relative expression of *G6PI1* and *PK1* significantly decreased at 48 h but significantly increased at 72 h (Figure 3), while *PK3* and *PK6* mRNA levels increased significantly at 48 h but decreased sharply (Figure 3). *PK5* mRNA level was significantly reduced at 48 h but had had little change at 72 h (Figure 3), and *PK10* mRNA level significantly increased at both 48 h and 72 h (Figure 3).

### 3.4 Relative expression of crucial enzyme genes in TCA-cycle

**FIGURE 4:**
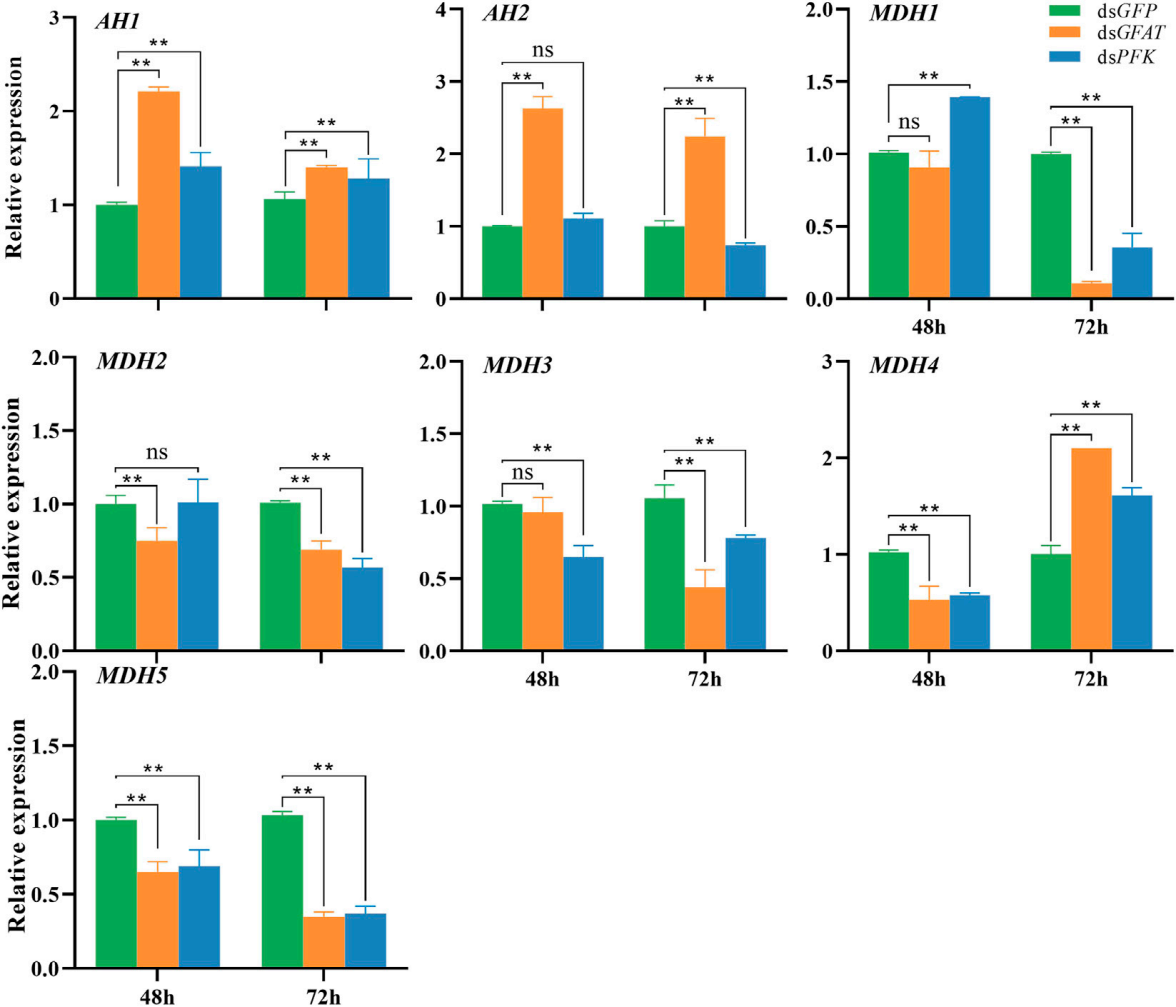
Relative expression of crucial genes in tricarboxylic acid cycle at 48 h and 72 h after dsRNA injection. The qRT-PCR is used to detect the genes expression levels and the 18 s RNA is used as internal control. Bars are means ± SE (standard error) of three biological replicates. An asterisk (*) represents significant differences (*p* < 0.05); two asterisk (**) represents extremely significant differences (*p* < 0.01). AH, aconitate hydratase; MDH, malate dehydrogenase.

Following the *GFAT* gene inhibition, the relative expression of *AH1* and *AH2* significantly increased at 48 h and 72 h (Figure 4), whereas the relative expressions of *MDH2* and *MDH5* were significantly decreased at these time points (Figure 4). Additionally, the relative expression of *MDH1* and *MDH3* had no significant change at 48 h but significantly decreased at 72 h (Figure 4), and the relative expression of *MDH4* dropped significantly at 48 h but increased at 72 h (Figure 4). After *PFK* gene inhibition, *AH1* expression was upregulated significantly at 48 h and 72 h (Figure 4), while the expression levels of *AH2* and *MDH2* only showed a slight decrease at 48 h but decreased significantly at 72 h (Figure 4). Moreover, the relative expression level of *MDH1* increased significantly at 48 h but decreased at 72 h (Figure 4), while the relative expression level of *MDH3* and *MDH5* decreased significantly at 48 h and 72 h (Figure 4). Lastly, the relative expression level of *MDH4* expression level was significantly downregulated at 48 h but upregulated at 72 h (Figure 4).

### 3.5 Enzyme activity of PK and MDH

**FIGURE 5:**
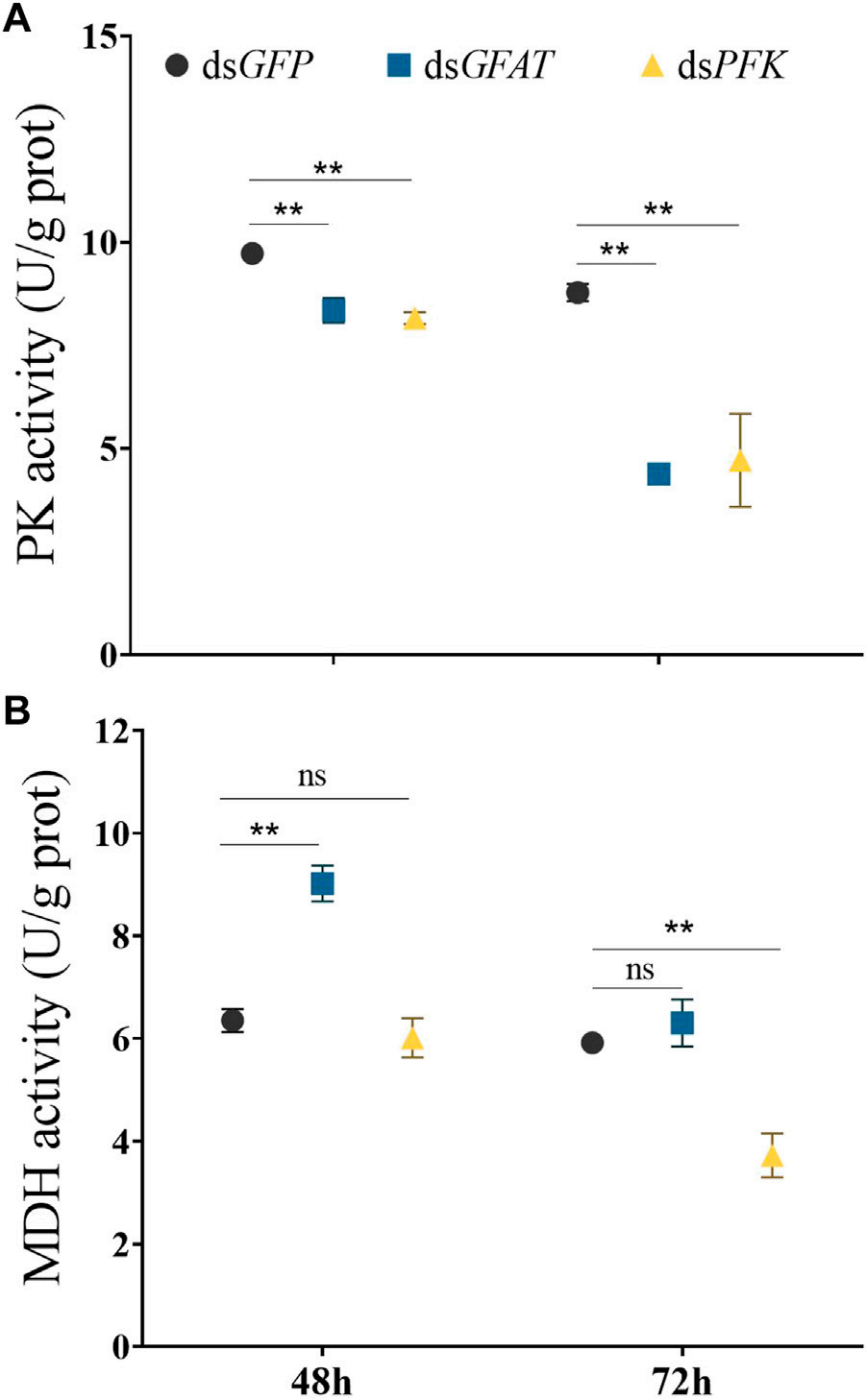
Pyruvate kinase activity and malate dehydrogenase activity at 48 h and 72 h after dsRNA injection. *Nilaparvata lugens* on 1st of 5th instar were used to inject. Bars are means ± SE (standard error) of three biological replicates. An asterisk (*) represents significant differences (*p* < 0.05); two asterisk (**) represents extremely significant differences (*p* < 0.01). ns: not significant.

Inhibition of *GFAT* significantly reduced PK enzyme activity at 48 h and 72 h, and MDH activity significantly decreased at 48 h (Figure 5). Similarly, *PFK* inhibition resulted in a significant reduction in PK enzyme activity at 48 h and 72 h, with MDH activity showing no significant change at 48 h but significantly decreasing at 72 h (Figure 5).

### 3.6 ATP content after dsRNA injection

**FIGURE 6:**
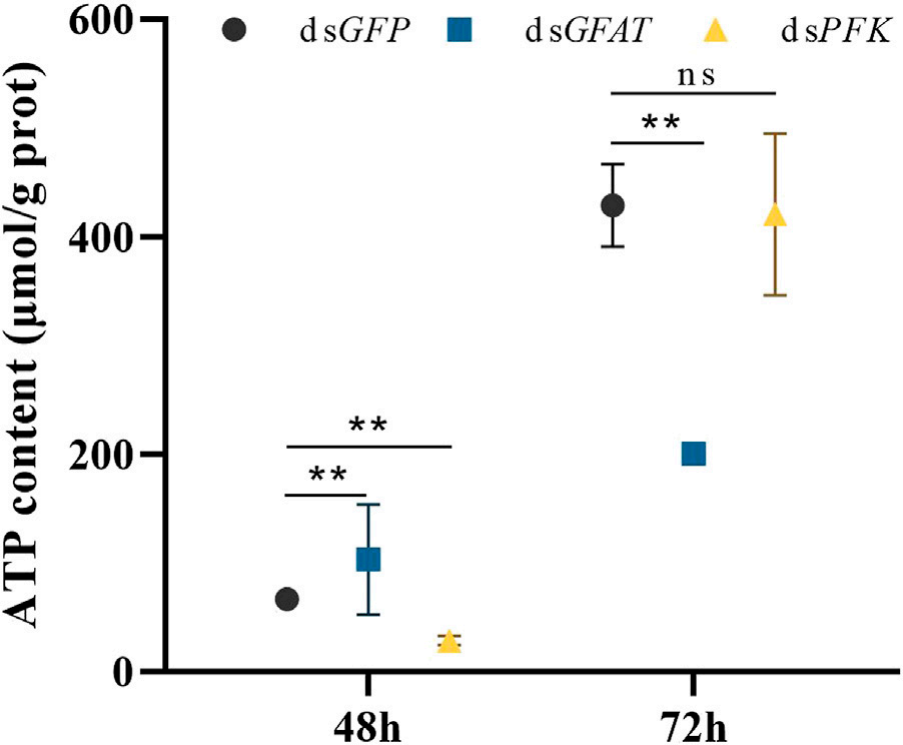
Content of adenosine triphosphate (ATP) at 48 h and 72 h after dsRNA injection. *Nilaparvata lugens* on 1st of 5th instar were used to inject. Bars are means ± SE (standard error) of three biological replicates. An asterisk (*) represents significant differences (*p* < 0.05); two asterisk (**) represents extremely significant differences (*p* < 0.01). ns: not significant.

Following ds*GFAT* injection, ATP content significantly increased at 48 h but decreased at 72 h after (Figure 6). However, after *PFK* inhibition, ATP content significantly extremely at 48 h but returned to normal levels at 72 h (Figure 6).

## 4 Discussion

GFAT is the rate-limiting enzyme for glucose flux through the hexosamine pathway. However, there have been few studies investigating the GFAT gene in insects, with only a limited numbers of species, including *Drosophila melanogaster*, *Aedes aegypti*, *Haemaphysalis longicornis*, and *Hyphantria cunea* (Graack et al., 2001; Kato et al., 2002; Huang et al., 2007; Zou et al., 2022). In contrast, PFK is the important rate-limiting enzyme in the glycolytic pathway (Jojima and Inui, 2015). AMP-activated protein kinase (AMPK) inhibits GFAT activity, thereby regulating the hexosamine pathway according to the organism’s energy requirements (Chang et al., 2000; Eguchi et al., 2009; Zibrova et al., 2017; Ruegenberg et al., 2021). In the even of starvation, cAMP-dependent protein kinase A can redirect glucose metabolism into energy production, rather than synthetic pathways that require hexosamines (Chang et al., 2000). AMPK also activates PFK through phosphorylation to stimulate glycolysis (Marsin et al., 2000), and the two enzymes play crucial roles in energy metabolism. Previous study has showed that *GFAT* and *PFK* genes exhibit contrasting regulation of chitin metabolism in BPH (Xu et al., 2021). However, their different effects on energy metabolism remain unknown.

UDP-N-acetylglucosamine (UDP-GlcNAc) is the final product in HBP and a substrate for O-linked glycosylation of cellular proteins (Qian et al., 2011). High glucose levels in cancer patients increase the flux into HBP and improve GFAT enzyme levels (Vasconcelos-Dos-Santos et al., 2017), resulting in increased GFAT activity and inhibited glucose uptake and glycogen synthesis in insulin-resistant patients (Srinivasan et al., 2007). However, knockdown of *NlGFAT* in our study caused little change in glucose content, but glycogen concentration significantly increased 48 h after ds*GFAT* injection leading to a metabolic shift where glucose is stored as trehalose (Seo et al., 2018). This suggests an inhibition of glycogen synthesis within a short period, possibly due to decreased glycogen degradation, as the expression levels of *NlGP* were also extremely significantly decreased within 72 h after ds*GFAT* injection. In addition, the expression of *NlTRE1-1*, *NlTRE1-2*, *NlTRE2*, *NlTPS1*, and *NlTPS2* also decreased extremely significantly simultaneously. Furthermore, the expression levels of *NlPPGM1*, *NlPPGM2*, *NlUGPase*, and *NlHK* significantly decreased at 48 and 72 h, indicating that BPHs can regulate sugar distribution by down-regulating the transcription levels of sugar metabolic enzyme genes after *NlGFAT* inhibition. *NlGFAT* expression was downregulated after inhibiting three *NlTRE* genes, while its expression was upregulated significantly at 72 h after *NlTPS1* and *NlTPS2* knockdown (Zhao et al., 2016; Yang et al., 2017). Despite the significant rise in glycogen content, the extremely significant decrease in *NlGS* expression at 48 and 72 h suggests that GS synthesis was inhibited, leading to the conversion of glycogen to trehalose (Seo et al., 2018). Additionally, NlG-6-pase expression significantly decreased at 48 and 72 h after *NlGFAT* knockdown, suggesting that glycogenolysis was also inhibited.

The expression of *NlTRE1-1*, *NlTRE1-2*, *NlTRE2*, *NlTPS1*, *NlTPS2*, *NlPPGM1*, *NlPPGM2*, *NlUGPase*, *NlGS*, *NlGP,* and *NlHK* were significantly downregulated after ds*PFK* injection. However, the changes in trehalose, glucose, and glycogen content were different from those observed with ds*GFAT* injection, suggesting that PFK may regulate the distribution of glucose (Gibb et al., 2017). Knocking down *NlPFK* resulted in little change to the trehalose content, but increased the glucose content and glycogen content after 72 h. Studies on cardiac myocytes suggest that when PFK activity was high, glucose uptake was increased, while glucose utilization is elevated, and when PFK activity was low, glucose uptake is minimally affected but glucose utilization is significantly reduced (Gibb et al., 2017). This could explain the significant increase in glucose content observed in our study, with some of the glucose being converted into glycogen for storage. The inhibition of key glycolytic enzymes favors gluconeogenesis (Belfiore et al., 1989).

Low PFK activity leads to a decrease in glycolysis metabolism (Gibb et al., 2017). The downregulation of *NlPFK* resulted in decreased expression levels of important enzyme in the glycolytic pathway and a sharp decrease in pyruvate kinase (PK) activity, which is in line with previous studies (Gibb et al., 2017). PK converts phosphoenolpyruvate and ADP to pyruvate and ATP in glycolysis, and pyruvate can be converted into acetyl-CoA, which combines with oxaloacetic acid to enter the TCA cycle (Israelsen and Vander Heiden, 2015). The TCA cycle, in combination with the subsequent electron transport chain, is one of the main metabolic pathways for providing energy under aerobic conditions (Gaster et al., 2012). The decrease of PK activity due to decreased *NlPFK* expression results in a decrease in pyruvate and subsequently a decrease in acetyl-CoA content. This decrease leads to the downregulation of enzyme genes in the TCA cycle, including *NLAH2*, *NlMDH1*, *NlMDH2*, *NlMDH3*, and *NlMDH5*, following ds*PFK* injection. Cytosolic MDH catalyzes the NADP-dependent oxidative decarboxylation of malate into pyruvate and carbon dioxide, generating NADPH. As an enzyme in production of NADPH, MDH is considered critical in TCA cycle (Farkas et al., 2002). Our results indicate that MDH activity also significantly decreased at 72 h after ds*PFK* injection. The decrease in enzyme genes expression in glycolysis-TCA cycle eventually led to an extreme decrease in ATP content 48 h after *NlPFK* knockdown.

According to Zhang et al. (2018), upregulated *GFPT2* in fibroblasts leads to less change in genes responsible for glycolysis, the pentose phosphate pathway, and TCA cycle. However, our results differ markedly from theirs. After inhibiting *NlGFAF*, the relative expression levels of *NlG6PI1*, *NlG6I2*, *NlPK1*, *NlPK2*, *NlPK3*, *NlPK4*, *NlPK5*, *NlPK7*, *NlPK8*, *NlPK9*, and *NlPK10* were significantly downregulated, and *NlPFK* expression also decreased significantly. Moreover, the PK activity also correspondingly decreased at 48 h and 72 h after ds*GFAT* injection. This suggested that the level of glycolytic metabolism also dropped sharply. However, the *NlAH1* and *NlAH2* expression increased extremely significantly after *NlGFAT* silencing, which is significantly different from ds*PFK* injection. Though the expression of *NlMDH1*, *NlMDH2*, *NlMDH3*, and *NlMDH4* also decreased significantly, the MDH activity increased extremely significantly at 48 h after ds*GFAT* injection, and the ATP content also increased extremely significantly at 48 h after *NlGFAT* knockdown, but decreased extremely significantly at 72 h. Thus, after *NlGFAT* was inhibited by RNAi, the level of glycolytic metabolism dropped sharply whereas the level of TCA cycle increased dramatically. In the study of mouse C2C12 muscle cells, despite profound suppression of both glucose and pyruvate oxidation, TCA cycle metabolism still were maintained, and TCA flux was achieved through enhanced reliance on glutaminolysis through malic enzyme and pyruvate dehydrogenase (PDH) as well as fatty acid and branched chain amino acid oxidation (Vacanti et al., 2014). This may be the reason that in our study, the level of glycolytic metabolism also dropped sharply whereas the level of TCA cycle increased dramatically. Unfortunately, we were unable to detect changes in fat and amino acid levels, but this still provides ideas.

In conclusion, the inhibition of *NlGFAT* or *NlPFK* resulted in a disorder of energy metabolism in BPHs. The knockdown of *NlGFAT* or *NlPFK* resulted in changes in distribution of trehalose, glucose, and glycogen and decrease of glycolysis. However, the downregulation of *NlGFAT* led to an increase in TCA cycle level and ATP content, whereas the downregulation of *NlPFK* caused a decrease of TCA cycle level and ATP content. Since NlGFAT and NlPFK influence the energy metabolism of BPHs, the development of corresponding enzyme inhibitors or activators might be explored for biological control of BPHs.

## Data Availability

The original contributions presented in the study are included in the article/supplementary material, further inquiries can be directed to the corresponding authors.
