# Erratum: Roles of *GFAT* and *PFK* genes in energy metabolism of brown planthopper, *Nilaparvata lugens*

**DOI:** 10.3389/fphys.2023.1286548

**Published:** 2023-09-13

**Authors:** Frontiers Production Office

**Affiliations:** Frontiers Media SA, Lausanne, Switzerland

**Keywords:** *Nilaparvata lugens*, RNA interference, glutamine, fructose-6-phosphate aminotransferase, phosphofructokinase, energy metabolism

Due to a production error, the **reference** for “Zhou et al. (2022)” was incorrectly written as “Zhou, C., Yang, X. B., Yang, H., Gong, M. F., Long, G. Y., and Jin, D. C. (2022). Geriatric early-stage triple-negative breast cancer patients in low-risk population: Omitting chemotherapy based on nomogram Sogatella furcifera Entomol. Gen. 42(5), 779–780. doi:10.1016/j.clbc.2022.08.013.” It should be “Zhou, C., Yang, X. B., Yang, H., Gong, M. F., Long, G. Y., and Jin, D. C. (2022). Role of insecticide-mediated transcription of the TOR and JH signaling pathway-related genes in the regulation of reproduction in Sogatella furcifera. Entomol. Gen. 42(5), 771–779. doi: 10.1127/entomologia/2022/1496.”

The publisher apologizes for this mistake. The original version of this article has been updated.

